# Mature tertiary lymphoid structures: important contributors to anti-tumor immune efficacy

**DOI:** 10.3389/fimmu.2024.1413067

**Published:** 2024-07-04

**Authors:** Xinyu Bao, Xuwen Lin, Mei Xie, Jie Yao, Jialin Song, Xidong Ma, Xin Zhang, Yinguang Zhang, Yiming Liu, Wenya Han, Yiran Liang, Hongling Hu, Li Xu, Xinying Xue

**Affiliations:** ^1^ Affiliated Hospital of Shandong Second Medical University, School of Clinical Medicine, Shandong Second Medical University, Weifang, China; ^2^ Department of Respiratory and Critical Care, Emergency and Critical Care Medical Center, Beijing Shijitan Hospital, Capital Medical University, Beijing, China; ^3^ Department of Respiratory and Critical Care, Chinese PLA General Hospital, Beijing, China; ^4^ Department of Thoracic Surgery, Beijing Tiantan Hospital, Capital Medical University, Beijing, China; ^5^ Department of Thoracic Surgery, Chinese PLA General Hospital, Beijing, China; ^6^ Department of Respiratory and Critical Care, Taihe Hospital, Hubei University of Medicine, Shiyan, China; ^7^ Department of Respiratory Medicine, Wuhan Central Hospital, Tongji Medical College, Huazhong University of Science and Technology, Wuhan, China; ^8^ Department of Respiratory Endoscopy, The Public Health Clinical Center Affiliated of Shandong University, Jinan, China

**Keywords:** mature tertiary lymphoid structure, germinal center, B cell, tumor immunotherapy, prognosis

## Abstract

Tertiary lymphoid structures (TLS) represent the ectopic aggregations of immune cells arising during chronic inflammation or tumor progression. In cancer, TLS are often associated with beneficial clinical outcomes in patients undergoing immunotherapy, underscoring their prognostic and predictive significance. Mature TLS, characterized by germinal centers and areas of T-cell and B-cell aggregation, are considered primary locations for activating and maintaining both humoral and cellular anti-tumor immune effects. Despite their recognized importance, the mechanisms driving the formation of mature TLS in cancer and their influence on the immune response within tumors remain insufficiently understood. Therefore, this review aims to comprehensively explore the structural composition, development mechanisms, maturity impact factors, immunological function, and innovative therapeutic strategies of mature TLS within the tumor microenvironment. The research summarized herein offers novel insights and considerations for therapeutic approaches to promote TLS generation and maturation in patients with cancer, representing a promising avenue for future cancer therapies.

## 1 Introduction

Over the past decade, immunotherapeutic interventions utilizing immune checkpoint inhibitors (ICIs) have exhibited notable therapeutic efficacy across a range of solid tumors, encompassing melanoma, triple-negative breast cancer, and non-small cell lung cancer (NSCLC) (1–3). In a recent phase 2 trial focused on surgically resectable NSCLC, neoadjuvant immunochemotherapy, when contrasted with neoadjuvant chemotherapy, significantly enhanced surgical feasibility, 24-month progression-free survival (PFS), and 24-month overall survival (OS) (4). Despite advances, current diagnostic markers for guiding ICI therapy, such as tumor mutational burden (TMB) and programmed death-ligand 1 (PD-L1)/programmed death-1 (PD-1) expression status, have limitations. PD-L1-negative or low-expressing NSCLC patients can benefit from immunocheckpoint inhibitor therapy (5–7). Additionally, the positive predictive effect of high TMB on ICI efficacy does not extend to all cancer types; some specific TMB-low cancers, including malignant gliomas, Merkel cell carcinomas, and Kaposi’s sarcomas, also respond positively to anti-PD-1 therapy (8–10). These observations indicate that PD-1 and TMB are not perfect predictive markers for immunotherapy efficacy, and they face challenges in accurately and comprehensively identifying the beneficiaries of immunotherapy. Thus, there remains an unmet need for reliable predictive biomarkers for immunotherapy in patients with cancer; identifying additional biomarkers is essential.

The presence of tertiary lymphoid structures within tumor tissue prior to treatment correlates with a favorable prognosis for patients, with increased TLS numbers associated with the effectiveness of neoadjuvant immunotherapy (11), which underscores the predictive and therapeutic significance of TLS in immunotherapeutic prognostication (12, 13). The prognostic value of TLS is influenced by their location, density, and maturity (14, 15). In particular, the first-line battleground for superior intra-tumoral anti-tumor immune responses is mature TLS with a well-defined structure and robust immune functionality. The maturation of TLS, marked by the formation of a germinal center (GC), signifies active anti-tumor immune responses, exhibiting a pronounced correlation with enhanced prognosis in cancer immunotherapy across a spectrum of solid tumors, including esophageal squamous carcinoma, bladder cancer, and pancreatic ductal carcinoma (16–18). Notably, active B cells within mature TLS contribute to potent humoral immunity, and also augment T cell-mediated immune responses. While mature TLS exhibit robust anti-tumor immune activity in cellular and humoral immune responses, the precise mechanisms governing their formation and functional pathways remain elusive.

In this review, we first elucidate the definition of mature tertiary lymphoid structures and describe the cellular composition of mature TLS. Next, we delve into the mechanistic aspects governing the formation of mature TLS and scrutinize their immunological functions. Finally, we propose the induction of mature TLS formation as novel tumor immunotherapy to provide innovative directions for clinical oncology treatment.

## 2 Definition of a mature tertiary lymphoid structure

Lymphoid tissues and organs serve as effective bastions against pathogenic invasion. However, in cases of persistent chronic infection, the body necessitates the establishment of a transient command center proximal to the site of infection to orchestrate rapid immune cell mobilization. This emergent lymphoid tissue, known as tertiary lymphoid structure, manifests as an ectopic aggregation of immune cells, evolving in response to sustained chronic inflammation, such as chronic infections, allograft rejection, and autoimmune diseases, where inflammatory signaling persists over extended periods (19–21). Remarkably, TLS are also a ubiquitous feature across most tumors, including NSCLC, melanoma, and colon cancer (22–24). The mature TLS comprise distinct compartments, including the high endothelial venule (HEV) expressing PNAd, a B-cell area, and a T-cell area. Within the T-cell zone, CD4+ T cells, CD8+ T cells, and mature dendritic cells (DCs) constitute the cellular constituents, while the B-cell zone predominantly harbors follicular dendritic cells (FDC) and B cells (11). Fibroblastic reticular cells (FRCs) are observed at the peripheries of the TLS. FDCs are primarily located within the core of the B-cell population and play pivotal roles in antigen presentation and B-cell activation. HEV facilitates lymphocyte recruitment and transit to sites of inflammation. In brief, the TLS serve as a strategic locale for localized immune cell interactions.

**Figure 1:**
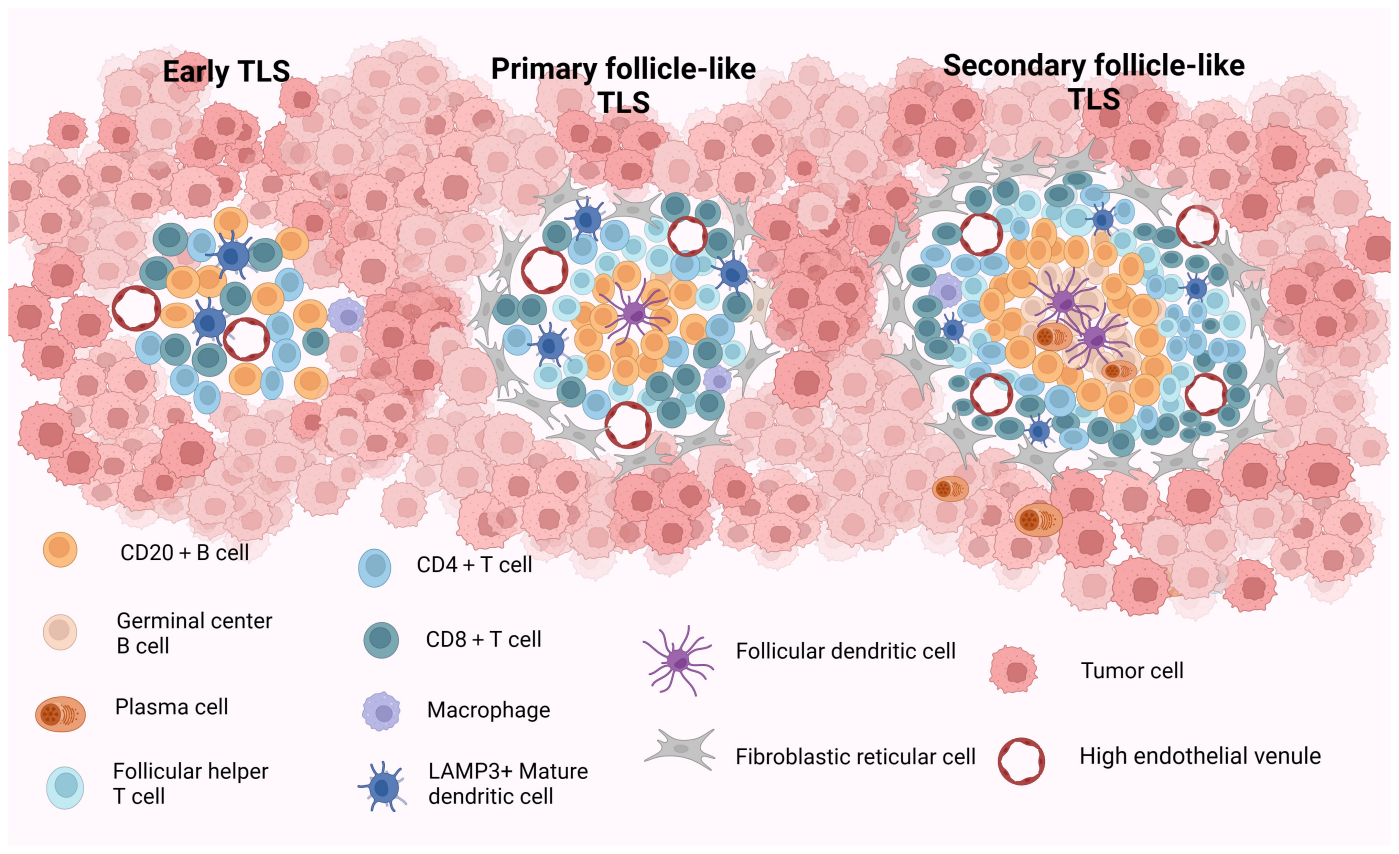
The structure and composition of TLS with different maturity. Early TLS (E-TLS) represent a dispersed cell aggregate comprising T and B cells along with stromal cells. Primary follicular-like TLS (PFL-TLS) are characterized by a B-cell zone containing CD21+FDC and a T-cell zone, lacking a germinal center reaction and exhibiting a denser lymphocyte arrangement. Secondary follicular-like TLS (SFL-TLS) consist of a B-cell zone housing proliferative germinal centers composed of B cells, plasma cells, and CD21+CD23+FDC, as well as a T-cell zone hosting DCs and Tfh cells. SFL-TLS display a pronounced germinal center reaction, with infiltrating lymphocytes being notably abundant and densely distributed within the TLS at this stage. HEVs are discernible across all three TLS forms, while FRCs are predominantly around PFL-TLS and SFL-TLS structures. TLS, tertiary lymphoid structures; E-TLS, early TLS; PFL-TLS, primary follicular-like TLS; SFL-TLS, secondary follicular-like TLS; HEV, high endothelial venule; DC, dendritic cell; FDC, follicular dendritic cell; Tfh, follicular helper T cell; FRC, fibroblastic reticular cell. Created with Biorender.com.

Current studies propose a staging system for the maturity staging of TLS, delineated as follows: (1) Early TLS (E-TLS), characterized by the T- and B-cell aggregates but no B-cell follicles and FDCs; (2) Primary follicle-like TLS (PFL-TLS), identified by the presence of a CD21+ FDC network within the B-cell area, lacking GC responses; and (3) Secondary follicle-like TLS (SFL-TLS), distinguished by the presence of a GC region containing CD21+CD23+ FDCs within the B-cell area. This configuration facilitates the selection of B cells possessing high-affinity B-cell receptors (BCRs) and fosters B-cell differentiation and activation (25–27). The structural and compositional features of TLS at various stages of maturity are described in **Figure 1**. Based on the aforementioned analysis, we deduce that truly mature TLS exhibit distinct yet adjacent zones enriched with T and B cells alongside activated FDCs within the B-cell compartments. Furthermore, mature TLS display evident B-cell class switching within the follicles, plasma cell (PC) differentiation, and conspicuous signs of GC responses (28). Briefly, mature TLS typically encompass CD21+CD23+ FDC networks and Ki67+CD23+ GC B cells (13, 29–31). Following prior research findings, we define secondary follicle-like TLS as representative of genuine manifestation of mature TLS in this review.

## 3 Main infiltrating cells of mature TLS

### 3.1 CD8+ T lymphocytes

CD8+ T lymphocytes serve as the principal effector phenotype of T cells, capable of recognizing and targeting tumor cell surface antigens, thereby eliminating them through the release of granzymes, perforins, and cytokines via diverse mechanisms (32). Given their potent tumor-killing capabilities, CD8+ T cells have long been a focal point in cutting-edge research on anti-tumor strategies. However, it is worth noting that although the proportion of TLS CD8+ T cells is relatively small, these cells predominantly localize to the periphery of PFL-TLS, exerting their significant anti-tumor effects primarily by migrating into the tumor stroma (23), suggesting that TLS CD8+ T cells play a pivotal role in anti-tumor immunity. A significant increase in the infiltration of neoantigen-reactive CD8+ T cells has been observed in the presence of mature TLS across various cancer types, exhibiting a positive correlation with patient survival. This indicates the TLS-mediated recruitment of CD8+ T cells, consequently augmenting CD8+ T cell-mediated anti-tumor responses, highlighting mature TLS as dominant loci of CD8+ T-cell immune activity (16, 33, 34). In patients with stage III lung adenocarcinoma undergoing adjuvant chemotherapy, the proportion of CD8+ CD103+ tissue-resident memory T cells (TRM) in TLS significantly increased with the maturation of TLS, with a more favorable prognosis linked to mature TLS displaying the CD103+ TRM high phenotype (35). Moreover, the secretion of the B-cell chemokine CXCL13 by chronically activated CD8+ T cells exposed to fibroblast-derived TGF-β has been shown to enhance B-cell spatial distribution and promote TLS maturation (36, 37). Additionally, a study identified antigen-sensitized, semi-clonal, activated, and differentiated tumor-infiltrating B cells (TIL-B) in gastric cancer, indicating their role primarily as antigen-presenting cells (APCs). The study also found that TLS exhibited markedly elevated mRNA expression levels for functional factors of CD8+ T cells, including CCL21, perforin, CXCL13, granzyme B (GZMB), and PD-L1, compared to non-TLS, suggesting that TLS are pivotal sites for T-cell cellular immunity (38). Another study demonstrated that tumors positive for mature TLS exhibited increased infiltration of CD8+ T cells, and the presence of TLS was significantly associated with improved objective response rates (ORRs) and OS in patients with high CD8+ T-cell density. Conversely, no similar correlation was observed in patients with low CD8+ T-cell density, indicating that sustained anti-tumor immune responses necessitate critical cooperation between T cells and mature B cells within TLS; the presence of CD8+ T cells alone may be insufficient (31). These findings underscore the supportive role of mature TLS in facilitating CD8+ T-cell activity, emphasizing the synergistic interactions between T cells and mature B cells within TLS. TLS represent a critical site for the activation and proliferation of CD8+ T cells, suggesting that despite their low abundance, CD8+ T cells may exhibit potent anti-tumor effects within the TLS. Further investigations are warranted to elucidate the precise contribution of CD8+ T cells to the overall anti-tumor efficacy of TLS.

### 3.2 Follicular helper T cells

Follicular helper T cells (Tfhs) constitute a specific subset of CD4+ T lymphocytes. Current literature predominantly defines authentic Tfh cells as CXCR5+ PD-1+ ICOS+ BCL6+ IL-21+ CD4+ T cells (39). Within TLS, Tfhs typically represent a prominent subpopulation within the T-cell compartment, commonly localized adjacent to B-cell compartments, with mature TLS displaying notable expression of Tfhs signature factors (40). Guided by specific chemokines, Tfhs migrate to the B-cell compartment, where they actively participate in GC responses and facilitate B cell-mediated immune responses, thereby playing roles in the maintenance of mature TLS (41, 42). In breast cancer, the active TLS have been correlated with the heightened expression of IL-21 and IFN-γ, secreted by Tfhs, suggesting that Tfhs drive the GC response within active TLS (43). Moreover, the differentiation of Ki-67+TIL-B was also found to be closely associated with the localization of CD4+ PD-1+ Tfhs within the GC, further validating the presence of active TLS. Collectively, Tfhs play a central role in both the establishment and functionality of mature TLS, particularly in regulating B-cell responses, GC reactions, and the sustainability of active TLS.

The favorable prognosis observed in immunotherapy associated with mature TLS may be intricately linked to Tfh cells. Across various cancer types, patients exhibiting pathological responses to ICI therapy showed significantly more Tfh and B-cell infiltration in tumor tissues compared to non-responders (44–46). These clinical benefits may stem from the modulation of PD-1 inhibition on Tfh cells by ICI treatment. Notably, pembrolizumab has been observed to bind to CD38+ Tfh-like cells within the TLS of bladder cancer, suggesting that Tfh cells represent a primary target of ICI within the CD4+ T-cell compartment (47). Given that the majority of Tfhs exhibit high PD-1 expression, PD-1-mediated inhibition can impair antigen-induced maintenance of Tfh cells, which conduce to limit Tfh cell numbers and prevent the production of low-affinity antibodies (48–50). Consequently, anti-PD-1 treatment may alleviate Tfh inhibition, thereby promoting differentiation of naïve CD4+ T cells into Tfh cells, consequently bolstering Tfh cell numbers and facilitating B-cell differentiation and antibody production (45). These observations are further corroborated in patients with melanoma undergoing ICI treatment, where increased B-cell differentiation and elevated IFN gene expression suggest an expansion in Tfh cell populations (51). Furthermore, Tfh-derived CXCL13 orchestrates the migration of CXCR5+ B lymphocytes to sites of chronic inflammation, thereby fostering TLS and GC formation (52). Highly differentiated Tfh cells have been shown to induce robust TLS formation and enhance B-cell recruitment. Conversely, reductions in Tfh cell populations result in compromised TLS, diminished immune infiltration, and weakened tumor control; however, these features can be restored following the transfer of pathogen-specific CD4+ T cells (53, 54). Tfh cells augment the effector function of CD8+ T cells through the secretion of IL-21 and IL-4, achieving a positive response to anti-PD-1 therapy (55, 56). Collectively, these findings underscore the critical role of Tfh cells not only in facilitating GC reaction and B-cell response but also in promoting CD8+ T-cell response, thus highlighting their potential as a crucial factor in enhancing immunotherapy efficacy.

### 3.3 B lymphocytes

Tumor-infiltrating B cells predominantly reside within TLS, primarily concentrated in the central region of mature TLS, where genes associated with GC B cells and plasmablasts are enriched. In contrast, the gene expression signatures of initial B cells are always absent (57). PCs may be found distributed throughout the interior or periphery of the TLS (58) (59). Characteristic B cells within mature TLS, identified as BCL6+, AID+, Ki67+, CD23+, and CD20+ GC B cells, exhibit distinctive molecular profiles of mature TLS. These B cells play a crucial role in TLS maturation, actively participating in the complete GC reaction, a critical process in TLS development. Within the GCs of mature TLS, B cells undergo selective activation, clonal expansion, somatic hypermutation (SHM), and class switch recombination (CSR) (51, 60). Subsequently, these B-cell clusters differentiate into plasma and memory B cells, generating antibodies specific to tumor-associated antigen (TAA) (16, 58). The heightened presence of B-cell memory features and PCs within mature TLS is associated with improved prognostic outcomes in cancer treatment, underscoring a significant humoral immune role for B cells within mature TLS (33, 51). In short, the unique structural characteristics of TLS make them prominent functional sites for B cells in the tumor microenvironment (TME).

The GCs within mature TLS serve as primary sites for B-cell proliferation and activation, generating robust humoral immunity. PCs and memory B cells within mature TLS contribute significantly to the potent humoral immune response and are strongly associated with favorable prognoses in immunotherapy. Research in NSCLC has revealed heightened PC signaling in tumors with TLS, leading to significantly increased OS following anti-PD-L1 therapy (59, 61). Several pivotal studies have also demonstrated that positive responses to tumor immunotherapy in patients with melanoma, renal cell carcinoma, and soft tissue sarcoma are linked to augmented B-cell infiltration and the presence of TLS (23, 26, 51). Furthermore, investigations have indicated that extended PFS in individuals receiving immunotherapy for head and neck squamous cell carcinoma is associated with enriched populations of overall B cells, GC B cells, or PCs. Transcriptomic analyses have corroborated these findings, revealing significant expression of B cell-related genes in patients who respond favorably to tumor immunotherapy (62, 63). These data underscore the significance of B-cell presence and function within mature TLS in promoting prolonged survival and improved treatment responses in patients.

### 3.4 Follicular dendritic cells

FDCs represent distinct non-hematopoietic stromal cells involved in the GC response; these cells are distinguished by their long surface dendrites and predominantly located within PFL-TLS and SFL-TLS within tumor environments (17, 31, 64). Although the precise origin of FDC progenitor cells remains elusive, activated local stromal cells have been observed to differentiate into FDCs following interactions with migrating immune cells within TLS (65, 66). FDCs in follicles facilitate the selection of high-affinity mature B cells primarily through antigen presentation to mediate TLS maturation. This process involves competitive binding of B cells to antigens displayed on the surface of FDCs, with those demonstrating optimal antigen binding receiving initial survival signals and subsequent coactivation signals from Tfh cells, thus promoting further B-cell affinity maturation (67). Moreover, FDCs possess the ability to bind and internalize nonhomologous immune complexes (ICs) derived from B cells via complement receptors 1 and 2. Upon internalization, these ICs are retained within non-degradable circulating compartments of FDCs and periodically presented on the cell surface, allowing for interaction with antigen-specific B cells to drive BCR affinity maturation and enhance B-cell specificity for antibodies (68). In addition to presenting antigens for GC B-cell selection, FDCs attract Tfh cells and B cells to form B-cell follicles by secreting CXCL13 and regulate T-cell activation by expressing PD-L1 and PD-L2 (69). Additionally, FDCs regulate IL-4 availability within GCs and facilitate the generation of memory B cells through IL-4Ra signaling (70). In summary, the immune function of FDC is an essential manifestation of the powerful anti-tumor immune effects of mature TLS.

As crucial components of mature TLS, FDCs significantly contribute to favorable prognoses by orchestrating the activation and maturation of B cells. One study demonstrated that activated B cells undergo proliferation and differentiation within mature TLS containing GCs characterized by the presence of CD21+CD23+ FDCs and Tfh cells, suggesting that comprehensive B-cell maturation occurs within this microenvironment (71). Furthermore, another investigation identified a network comprising CD23+ FDCs, PNAd+ HEVs, and BCL6+CD20+ B cells within GC-positive TLS in renal carcinoma, considering these distinctive cells and structures as indicative markers of mature TLS. Remarkably, a substantial correlation was observed between these markers and the densities of IgG+ and IgA+ PCs (57). Notably, patients harboring SFL-TLS with CD21+CD23+ FDCs exhibited more favorable prognoses in immunotherapy compared to those with PFL-TLS containing only CD21+ FDCs (25, 28), potentially attributed to enhanced BCR diversity within SFL-TLS, along with increased plasma and memory cell differentiation and the presence of GC responses (72), which highlights the differential functionality of FDCs in these two TLS types, suggesting that while FDCs may activate B cells effectively within SFL-TLS, their activity may be diminished or insufficient within PFL-TLS. These findings underscore the significance of FDC-mediated activation of B-cell proliferation and differentiation within mature TLS, contributing to the high density of PC infiltration observed in tumors hosting mature TLS. Additionally, FDCs within TLS can secrete CXCL13 to attract B cells towards the tumor site, thereby promoting the formation and sustenance of TLS. Based on the above studies, it is rational to speculate that functional FDC may be the dominant factor that differentiates mature TLS from immature TLS in terms of anti-tumor immune efficiency.

## 4 Specific mechanisms and factors influencing the formation and maturation of tertiary lymphoid structures

### 4.1 Exploration of the mechanisms of tertiary lymphoid structure formation and maturation

**Figure 2:**
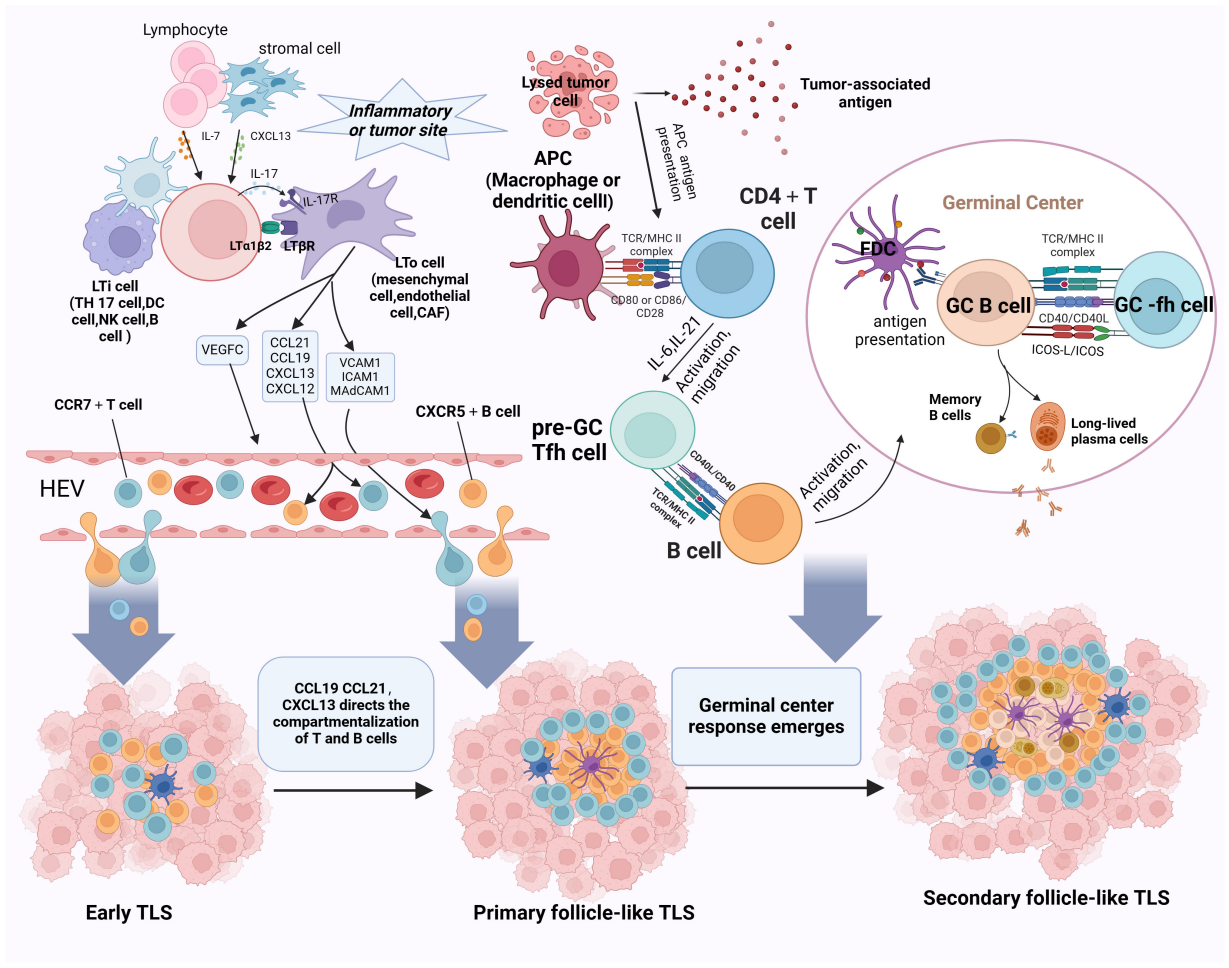
Schematic representation of the progression of TLS development and maturation. The initiation of TLS formation commences with the release of CXCL13 and IL-7 by stromal cells, which prompts the recruitment of LTi cells into tumor tissues. B cells, Th17 cells, NK cells, and DC cells take on the role of LTi cells. CAFs, mesenchymal cells, and endothelial cells substitute for LTo cells. Paracrine signaling of IL-7 induces LTi cells to express Tα1β2 and bind to LTβR on activated LTo cells. This LTβR signaling stimulates LTo cells to secrete VEGFC, consequently facilitating HEV formation. Additionally, IL-17 secreted by LTi cells binds to IL-17R on LTo cells. The LTα1β2-LTβR and IL-17-IL-17R signaling pathways promote the secretion of adhesion molecules (MADCAM1, ICAM1, and VCAM1) and chemokines (CXCL12, CXCL13, CCL19, and CCL21) by LTo cells, attracting lymphocytes to infiltrate tumor tissues via HEVs, thus initiating the formation of E-TLS. Subsequently, CCL19/CL21 and CXCL13 orchestrate the compartmentalized distribution of CCR7+ T cells and CXCR5+ B cells, respectively, leading to the organized arrangement of TLS and the transition to the primary follicle-like TLS stage. Finally, with the assistance of FDCs and Tfh cells, the germinal center reaction ensues, facilitating SHM, B-cell clonal selection, and the generation of high-affinity antibodies within the TLS. This maturation process culminates in the progression to SFL-TLS. LTi, lymphoid tissue-inducing cell; LTα1β2, lymphotoxin α1β2; LTβR, lymphotoxin β receptor; VEGFC, vascular endothelial growth factor C; CAFs, cancer-associated fibroblasts; SHM, somatic hypermutation. Created with Biorender.com.

Considering the excellent anti-tumor immune capabilities of mature TLS, investigating the formation mechanisms of mature TLS offers valuable insights for interpreting their functionality and devising strategies to induce TLS formation. This paper will explore the developmental and maturation mechanisms of TLS along the general developmental pathway, encompassing the progression from early TLS to primary follicle-like TLS to secondary follicle-like TLS (refer to **Figure 2**).

At the onset of the TLS formation, stromal cells or lymphocytes present at sites of chronic inflammation or tumor secrete chemokines such as CXCL13 and IL-7, thereby attracting lymphoid tissue-inducing (LTi) cells to the tumor site; B cells, Th17 cells, and M1 macrophages act as potential LTi cell candidates. Cancer-associated fibroblasts (CAFs), mesenchymal cells, and endothelial cells substitute for lymphoid tissue organizer (LTo) cells. Paracrine signaling of IL-7 induces LTi cells to express lymphotoxin α1β2 (LTα1β2) and bind to lymphotoxin β receptor (LTβR) on activated LTo cells. This LTβR signaling stimulates LTo cells to secrete vascular endothelial growth factor C (VEGFC), consequently facilitating HEV formation. Notably, differing from an LTβR-dependent pathway, M1 macrophages function as LTi cells via the TNF-α/TNFR axis between M1 macrophages and LTo cells (73). Additionally, IL-17 secreted by LTi cells binds to IL-17R on LTo cells. Ultimately, the LTα1β2/LTβR, TNF-α/TNFR, and IL-17/IL-17R signaling pathways promote the secretion of adhesion molecules (MADCAM1, ICAM1, and VCAM1) and chemokines (CXCL12, CXCL13, CCL19, and CCL21) by LTo cells. These molecules attract additional stromal and hematopoietic cells into the tumor and recruit lymphocytes via HEVs, initiating early TLS formation (12, 74–76).

The progression of TLS development entails the segregation of homeostatic chemokines, which establish compartmentalized zones of nascent lymphoid follicles. This process induces spatial structural alterations in the TLS, characterized by CCL19+ and/or CCL21+ FRCs and CXCL13+ FDCs. CCL19+ and CCL21 orchestrate the spatial distribution of CCR7+ T cells, and CXCL13 directs the spatial distribution of CXCR5+ B cells, ultimately forming distinct T- and B-cell zones (52, 77). Through this mechanism, the TLS transitions into the primary follicle-like TLS stage. Notably, FDCs, which are non-migratory and essential components of PFL-TLS, pose a lingering question regarding their origin within TLS. Although some studies have proposed that FDCs in secondary lymphoid organs may derive from perivascular mural cells (78), the specific source of FDCs in TLS remains unknown. It has been suggested that localized cellular responses trigger inflammation in the vascular endothelium, attracting membrane-bound LTα1β2 LTi to the site of inflammation. These cells then bind to LTβR on LTo cell surfaces, leading to LTo cell amplification and CXCL13 secretion. CXCL13, in turn, attracts B cells transvascularly to the site of FDC development (79). Additionally, LT and TNF-α secreted by B cells stimulate perivascular mural cell proliferation and upregulate Mfge8/CXCL13 expression (80). In the presence of B cells, pre-FDCs undergo further development into functional FDCs expressing CD21/35 and FcgRIIb, gaining the ability to capture ICs (78, 81). However, whether this proposed mechanism of FDC development applies to the evolution of early TLS to primary follicle-like TLS remains unclear.

As primary follicle-like tertiary lymphoid structures progress, B cells within the TLS internalize and process novel antigens, presenting them to helper T cells and receiving co-stimulatory signals. Subsequently, B cells competitively receive signals from Tfh cells, and positively selected B cells will enter into the dark zone, differentiate into PCs, or do further clonal expansion and SHM. After multiple rounds of expansion and SHM, the affinity of BCRs is enhanced. Subsequently, a significant influx of B cells with diverse affinities migrates to the GC light zone, competing for binding to the limited antigen presented on FDCs. B cells loaded with abundant antigens have heightened opportunities to bind to Tfh cells in the light zone, thus receiving co-activation signals vital for B-cell selection. This process heavily relies on the CD40L–CD40 interaction between Tfh cells and B cells. Surviving B cells return to the dark zone of the GC, where they undergo repeated rounds of cell proliferation and SHM to further refine their BCR affinity. Conversely, some B cells differentiate into plasma or memory cells and exit the GC, while those failing to receive activation signals from FDCs are prone to apoptosis (66, 82). This progression coincides with the transition from CD21+ FDCs in the PFL-TLS stage to CD21+CD23+ FDCs in the SFL-TLS stage, concomitant with B-cell differentiation and maturation. Notably, heightened CD23 expression is pivotal for FDC activation and maturation, facilitating their integration into GC responses (78). Mature FDCs exhibit increased surface expression of adhesion molecules and Fc receptors, enhancing their antigen-capture capacity (80, 83). However, the precise mechanisms governing the molecular alterations in FDCs remain elusive. As memory B cells and PCs emerge and high-affinity antibodies are generated, TLS progress toward a secondary follicle-like phase. We postulate that the phenotypic shift in FDCs constitutes a crucial aspect of TLS maturation, yet the specific mechanisms driving the transition from primary to secondary follicular TLS remain unidentified.

Given the considerable structural and functional similarity between TLS and secondary lymphoid organs (SLOs), many current insights into TLS formation and maturation are extrapolated from studies centered on SLOs. However, disparities in fine structure and tumor immune microenvironment (TIME) between TLS and SLOs may constrain the direct application of SLO formation mechanisms to TLS. Additionally, our understanding of the cellular and molecular mechanisms orchestrating TLS formation predominantly originates from models of autoimmune diseases and chronic infections. Hence, while these mechanisms offer valuable insights, they should be regarded as references rather than definitive guidelines for understanding TLS formation in the context of cancer.

### 4.2 Factors influencing the development and maturation of tertiary lymphoid structures

Besides the elusive process of TLS evolution towards maturity, the determinants influencing TLS formation and maturation remain poorly understood. Ongoing investigations suggest that various factors may contribute to TLS maturation, including bacterial infections, tumor pathological characteristics, tumor type, TLS location, treatment regimen, and tumor-draining lymph nodes (TDLNs). For instance, analysis of single-cell RNA-Seq data from human gastric tissues reveals that *Helicobacter pylori*-infected stomachs tend to exhibit activated phenotypic B cells implicated in the formation of mature TLS (84). Furthermore, neuroendocrine-differentiated gastric cancers manifest a higher abundance of less mature TLS compared to those lacking neuroendocrine attributes, with a high infiltration frequency of naïve B-cells, regulatory T cells, and exhausted CD8+ T cells, and elevated PD-L1 expression (85). The cancer type may also impact TLS organization; investigations into TLS immunophenotypes in bladder and renal cell carcinomas indicate a higher likelihood of detecting mature TLS with GCs in bladder cancer. Moreover, discrepancies in the TIME surrounding TLS are observed between the two tumors; compared to renal cancer, TLS in bladder cancer are characterized by a heightened proportion of CD8+, FOXP3+, and PD-L1+ cells (18). Moreover, the maturation of TLS could be influenced by TDLNs, where diminished IFN-γ signaling from B cells and natural killer (NK) cells leads to reduced infiltration of memory B-cell populations in the TDLNs and subsequently hampers the transportation of B cells from TDLNs to the primary tumor site, thus impeding TLS maturation within the tumor (86). Neoadjuvant chemotherapy has been shown to compromise TLS maturation and lead to GC loss (29). Furthermore, observations reveal a discrepancy in TLS distribution based on tumor location; for instance, left colon cancers exhibit a higher prevalence of early-stage TLS, whereas right colon cancers are more inclined towards follicular TLS, indicating a potential correlation between TLS maturity and tumor site (24). In clear cell renal cell carcinoma, E-TLS and PFL-TLS are predominantly found in the distal region of the tumor, characterized by elevated frequencies of PD-L1-overexpressing tumor-associated macrophages and regulatory T-cell infiltration. Conversely, SFL-TLS is primarily distributed in the proximal tumor region (87). Notably, melanoma cases feature fully developed TLS with Ki67+ AID+ B cells predominantly in skin metastases, whereas FDCs are identified in select lung, muscle, and intestinal metastases but are absent in brain metastases, hinting at a potential association between TLS maturity and metastatic organ site (88). In summary, TLS maturation are subject to multifaceted influences. Future research endeavors should delve deeper into the underlying factors affecting TLS formation and maturation, thereby enriching the theoretical framework for TLS-induced immunotherapy.

## 5 Immunological functions of mature TLS associated with favorable prognosis for immunotherapy

**Figure 3:**
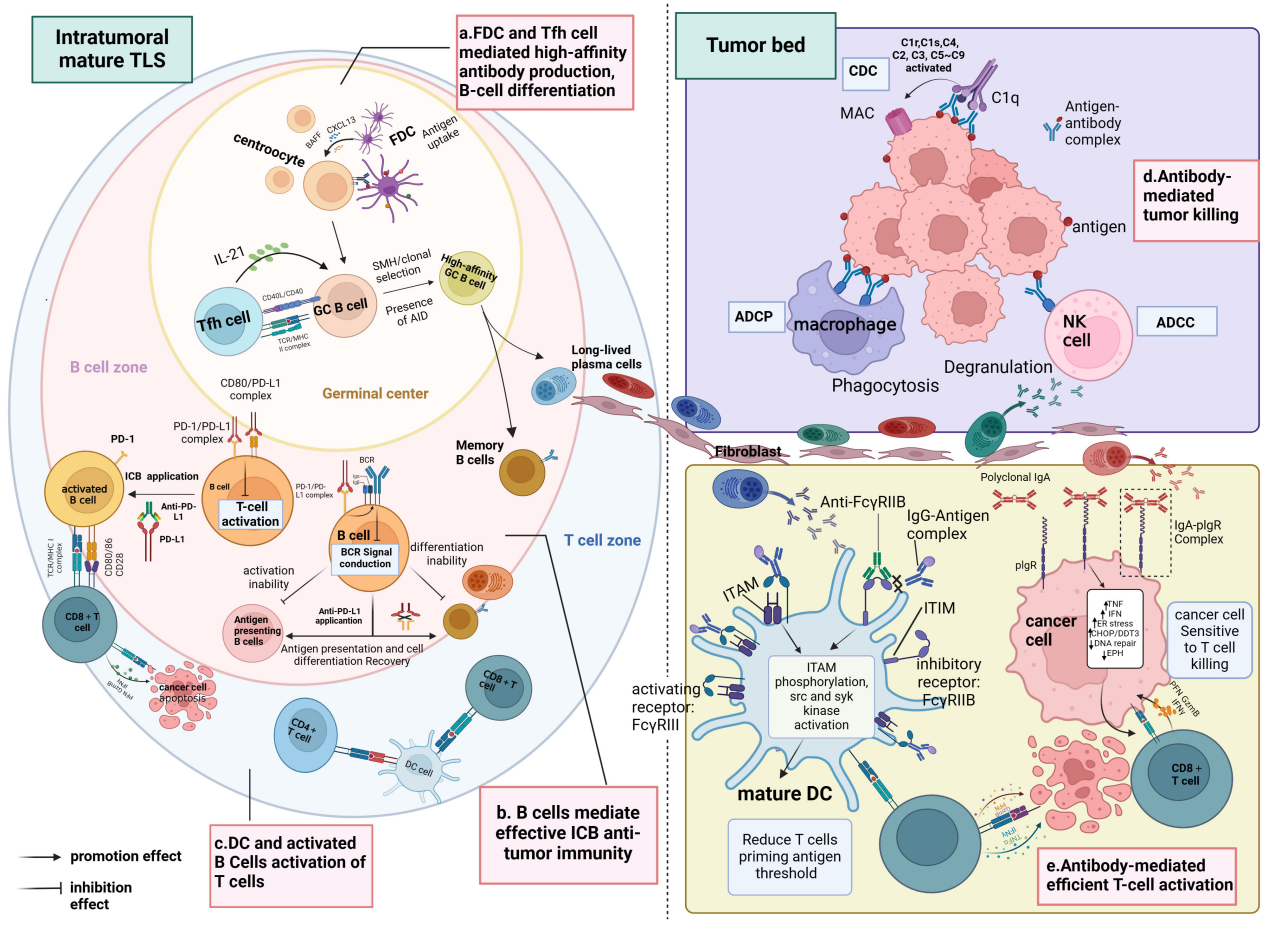
Immunological functions of mature TLS. **(A)** Within the SFL-TLS, B cells interact with FDCs and Tfh cells, stimulating B cells to undergo clonal expansion, SHM, clonal selection, and differentiation into antibody-producing plasma cells and memory B cells. Plasma cells migrate from the TLS to the tumor bed along the fibroblast track and produce tumor-associated antibodies. **(B)** Anti-PD-L1 therapy can disrupt the binding of PD-L1 to CD80, thus relieving the inhibition of BCR signaling imposed by the PD1/PD-L1 complex on B cells, leading to B-cell activation and enabling more efficient antigen presentation to T cells; this process also promotes the differentiation of B cells into plasma cells and memory B cells. Activated B cells liberated from ICI serve as APCs to present TAAs and activate T cells. **(C)** DCs positioned within the T-cell zone of the TLS contribute to antigen presentation and the activation of both CD4+ and CD8+ T cells. **(D)** Antibodies exert their anti-tumor effects through the ADCC and ADCP pathways mediated by macrophages and NK cells. In the CDC pathway, antibodies can bind to complement C1q, initiating a classical complement activation cascade (C2–C9), which culminates in forming membrane-attacking complexes that lyse cancer cells.**(E)** Mature DCs play a pivotal role in eliciting antigen-specific effector T-cell responses through antigen presentation. Complexes of IgG and antigen peptides selectively bind to both activating (FcγRIII) and inhibitory (FcγRIIB) receptors on the surface of DCs, modulating DC maturation. Interaction with activating FcγRIII triggers the ITAM, leading to downstream activation responses mediated by src and syk kinases. Conversely, engagement with inhibitory FcγRIIB activates the ITIM, which counteracts ITAM-mediated activation signals. By blocking FcγRIIB, ICs preferentially activating FcγRIII, promoting DC maturation and initiating T-cell responses. Moreover, binding of antibody IgA to the PIGR on the tumor cell surface induces transcriptional alterations, including upregulation of tumor cell inflammatory pathways (TNF signaling and IFN-γ receptor signaling), endoplasmic reticulum stress-related genes (CHOP/DDIT3 signaling), and pro-apoptotic genes, while downregulating DNA repair pathways and genes associated with tumor progression (such as EPH). These changes enhance the susceptibility of tumor cells to attack by CD8+ T cells. BCR, B-cell receptor; PD-1, programmed death-1; PD-L1, programmed death-ligand 1; ICI, immune checkpoint inhibition; APC, antigen-presenting cell; TAA, tumor-associated antigen; MHC, major histocompatibility complex; TCR, T-cell receptor; DC, dendritic cell; ADCC, antibody-dependent cellular cytotoxicity; ADCP, antibody-dependent cellular phagocytosis; IgA, immunoglobulin A; IgG, immunoglobulin G; ITAM, immunoreceptor tyrosine-based activation motif; ITIM, immunoreceptor tyrosine-based inhibition motif; IC, immunocomplexes; PIGR, polyimmunoglobulin receptor. Created with Biorender.com.

These studies collectively converge on the conclusion that tumors characterized by mature TLS, high densities of B cells, PCs, tumor-reactive T cells, and antibodies targeting TAA typically exhibit favorable clinical outcomes and display effective responses to immunotherapy compared to tumors lacking these features. This phenomenon may be attributed to the significant influence of TLS maturity on the functional status and distribution of B cells within the TME, where infiltrating B cells within immature TLS tend to be naïve and inactive. Furthermore, additional research indicates a positive correlation between TLS maturity and the level of CD8+ T-cell infiltration into the tumor mesenchyme, suggesting that tumors harboring mature TLS may benefit from immunotherapy owing to heightened infiltration of activated CD8+ T cells in the mesenchyme. In essence, mature TLS foster an advantageous spatial environment conducive to B-cell maturation and differentiation, as well as the activation of CD8+ T cells, thus emerging as a critical hub within the TIME for orchestrating superior anti-tumor immune responses (refer to **Figure 3**).

### 5.1 The germinal centers of mature TLS are microanatomical structures in which B-cell receptor mutations and antibody affinity mature

The GC arises from the extensive proliferation of antigen-specific B cells upon activation, marked by activation-induced cytidine deaminase (AID), which initiates processes like SHM and CSR to modulate immunoglobulin affinity and maturation. B cells typically undergo SHM and CSR within GCs, where they are regulated by FDCs and Tfh cells to differentiate into memory B cells and long-lived PCs capable of producing high-affinity antibodies, critical for executing B cell-mediated immune responses. Noteworthy is the prevalence of GCs within intra-tumoral mature TLS, with recent studies elucidating the sequential maturation stages leading to GC formation within small cell lung cancer TLS (60).

Numerous studies have documented GC responses within TLS tumors. For instance, in NSCLC, Germain et al. identified AID expression in GC-positive TLS, a pivotal enzyme facilitating B-cell SHM, CSR, and immunoglobulin gene conversion (89). Multiple research cohorts have observed that the characterization of BCR libraries aligns with antigen-driven clonal expansion, with SHM levels correlating with survival among tumor-associated B-cell (TAB) populations (90–93). Consistent findings indicate a positive association between increased mature TLS counts and B-cell expansion, heightened BCR diversity, augmented frequencies of memory B cells, plasmablast/PC-like B cells, and activated CD69+ B cells in patients with melanoma (23, 51). Moreover, a study on breast tumors by Liu et al. revealed distinctions in surface marker expression between intra-tumoral B cells and peripheral blood B cells; the expression of co-stimulatory proteins such as CD86 was found to be upregulated, whereas the expression of CD23 was downregulated in intra-tumoral B cells, and CD23 is a negative regulator of B-cell activity and was downregulated in mature B cells that undergone SHM and CSR (94). Notably, investigations into renal cell carcinoma indicated that in tumors harboring dense TLS and PCs, PCs were predominantly distributed around the B-cell follicles, synthesizing IgG or IgA antibodies capable of binding to tumor cells, indicating that PCs in the tumor can produce anti-tumor antibodies *in situ* (57), corroborating findings from studies on soft tissue sarcoma and NSCLC (58, 89, 95). Pancreatic ductal adenocarcinomas harboring TLS exhibit an elevated presence of memory B cells compared to tumors lacking TLS. Within the TME, the presence of TLS correlates with a decrease in IgD+ and IgM+/IgD+ naive B cells relative to peripheral blood, suggesting potential antibody class switching within the TLS (28). Mature TLS display an augmented proportion of B-cell memory characteristics and PCs, which produce a highly selective antibody repertoire, activate the complement pathway, and initiate antibody-dependent cytotoxicity (ADCC), thereby enhancing the anti-tumor effects of TLS (96). In conclusion, a series of studies indicate that within the GCs of mature TLS, processes including B-cell selection, activation, antibody affinity maturation, antibody class switching, and PC differentiation occur, resulting in the local generation of anti-tumor antibodies. Mature TLS thus serve as an immunologically active site for anticancer B cells (53, 97, 98).

### 5.2 B cells act as antigen-presenting cells to activate T cells and promote T-cell anti-tumor responses

Mature TLS host a higher abundance of functionally activated B cells, characterized by elevated levels of major histocompatibility complex (MHC) class II and B7 molecules on their surface compared to B cells within immature TLS, facilitating enhanced antigen presentation and T-cell activation (99). Specifically, B cells uptake and process tumor-released antigens, generating antigenic peptide–pMHC complexes. Co-stimulatory molecules on B-cell surfaces interact with corresponding receptors on T cells, providing crucial co-stimulatory signals for dual T-cell activation, thereby augmenting T-cell immune responses (100). B cells function as specialized APCs proficient in processing MHC class I and class II antigens, leading to their presentation to CD8+ and CD4+ T cells, thereby fostering T-cell priming and activation (101). Single-cell transcriptome analysis of melanoma samples unveiled a transcriptional upregulation of CD40LG and CD83 in Tfh and B cells within mature TLS, respectively. Moreover, Cellchat analysis delineated interactions within the MHC I/MHC II pathway between B cells and CD8+ T cells/CD4+ T cells, suggesting antigen presentation and subsequent T-cell activation orchestrated by B cells. Consistent with these observations, the study further demonstrated that enhanced B-cell infiltration and increased BCR diversity following ICI therapy facilitated B-cell presentation of diverse tumor antigens, thereby fostering the activation of Tfhs and CD8+ T cells post-immunotherapy (102). Furthermore, in colorectal and gastroesophageal cancers, a substantial presence of CD86+ B cells has been reported, primarily localized within the lymphoid follicles of the TLS and exhibiting a positive correlation with TLS abundance. These B cells demonstrate the capacity to elicit T-cell responses against cancer-testosterone antigens *in vitro* (103). Analogous findings emerged from a study of human papillomavirus (HPV)+ oropharyngeal squamous cell carcinoma, revealing a positive correlation between the density of CD20+ TIL-B interacting with CD8+ tumor-infiltrating lymphocytes (TILs) and the infiltration frequency of HPV-specific CD8+ T cells (104); the density of B-/T-cell interactions was further associated with a favorable prognosis for immunotherapy, with B-cell exhaustion following anti-CD20 immunotherapy resulting in diminished tumor infiltration by macrophages and CD8+ T cells (105). In conclusion, TLS within TME serve as the primary niches for B cells and offer sustained assistance for activating T cells.

PCs may mediate effective antigen presentation by DCs to T cells by secreting antibodies. Studies on ovarian cancer have identified a correlation between PCs and heightened levels of CD8+, CD4+, and CD20+ TILs, alongside the expression of various cytotoxicity-associated gene products. Moreover, the therapeutic benefits of CD8+ TILs are observed only in the presence of PCs, underscoring the collaborative synergy between T and B cells in bolstering anti-tumor immunity (58). Notably, B cells and PCs preferentially express immunoglobulin A (IgA), which can be internalized by tumor cells via binding to the polymeric immunoglobulin receptor (PIGR) in an antigen-independent manner, rendering tumor cells more susceptible to T cell-mediated cytotoxicity (106, 107). Furthermore, research by Kalergis et al. has elucidated that ICs can selectively bind to agonistic Fcγ receptors (FcγRIII) on DCs, promoting DC maturation and subsequent T-cell activation, thereby amplifying T-cell responses (108). These findings collectively suggest that therapies aimed at enhancing humoral immunity may offer superior efficacy compared to interventions merely targeting T cells, particularly in malignancies exhibiting resistance to checkpoint inhibitors.

### 5.3 Plasma cell-derived antibodies participate in anti-tumor immune responses as activators of acquired immunity

PCs are predominantly clustered around mature TLS, constituting approximately 90% of the tumor stroma (58, 109). In ovarian cancer, the upregulation of an autoantigen, matrix metalloproteinase 14, has been identified as a driver of B-cell activation and subsequent autoantibody production within TLS (110). Furthermore, PC aggregations are observed near TLS and adjacent to follicle-centered B cells. Concurrently, IgG, IgM, and IgA deposits on tumor cells, while notably absent in healthy tissues (111), suggest the local production of antibodies within TLS. This phenomenon is associated with prolonged survival in patients with tumors rich in TLS (112). Functionally, these antibodies exert anti-tumor effects both directly, targeting specific antigens on tumor cells, and indirectly through mechanisms such as ADCC, antibody-dependent cellular phagocytosis (ADCP), and complement-dependent cytotoxicity (CDC). Subsequently, NK cells and macrophages eliminate dying tumor cells, releasing tumor antigens that DCs can capture in the peritumoral region. Antigen-presenting DCs then activate tumor-infiltrating T and B lymphocytes or stimulate CD4+ Tfh cells within TLS, initiating the cascade of B-cell proliferation and differentiation.

Several studies suggest that antibodies exert tumor-killing effects through ADCC, although direct evidence supporting this theory remains elusive (113, 114). Analysis of The Cancer Genome Atlas (TCGA) data through bioinformatic approaches revealed a positive correlation between B-cell characteristics and the expression of Fc fragments of IgG receptor IIIa (FCGR3A) and GZMB, indicating potential involvement of ADCC mediated by NK cells (115). In human renal cell carcinoma, enrichment of IgG and apoptotic cells around TLS is often accompanied by infiltration of CD68+ myeloid cells, which are typically positioned near apoptotic tumor cells; this suggests an indirect implication that TIL-B-derived antibodies induce ADCP to eliminate tumor cells (57). Colorectal cancer-related research demonstrated that tumor-infiltrating macrophages exhibit phagocytic activity against tumor cells in the presence of anti-tumor IgG *in vivo*. Additionally, *in vitro* experiments highlighted that the uptake of ICs by DCs is sufficient to initiate anti-tumor T-cell responses (116). Notably, recent studies in a large cohort of patients with ovarian cancer revealed that polyclonal IgA antibodies derived from TABs bind to IgA receptors on tumor cells, triggering pIgR-mediated IgA transcytosis and impeding tumor growth, ultimately enhancing tumor cell killing by T cells (106). Collectively, these findings indirectly suggest that PC antibodies mediate anti-tumor immune responses through pathways involving ADCC, ADCP, and other mechanisms.

### 5.4 Follicular helper T cells of mature TLS are important participants in B-cell activation

The potential anti-tumor mechanism of mature TLS involves the synergistic interaction between Tfh cells and B cells. Immunofluorescence imaging of TLS confirmed the spatial co-localization of Tfh and B cells within GCs, supporting T cell–B cell interactions. This interaction is facilitated by surface co-stimulatory molecules such as “ICOSL-ICOS” and “CD40-CD40L,” which support T–B cell crosstalk. Subsequently, B cells undergo proliferation and differentiation into memory B cells and PCs, significantly enhancing the anti-tumor efficacy of SFL-TLS (52, 117). Furthermore, studies have elucidated that CD40 on the surface of Tfh cells binds to CD40L on GC B cells, promoting positive selection of B cells. Under the influence of various cytokines (TGF-β, IFN-γ, IL-4, IL-21, and IL-6), Tfh cells support the development of GC B cells into memory or PCs, as well as facilitating processes such as SHM and antibody CSR (55, 118, 119). The development of GCs is intricately linked to the participation of Tfh cells, which drive the differentiation of B cells into long-lived PCs and high-affinity memory cells (120, 121). B cells that have not undergone cellular differentiation can re-enter the GCs during further SHM, leading to further maturation of BCR affinity (122). Transcriptomic analysis based on TCGA data reveals elevated expression levels of MAF and CD200, markers associated with Tfh cells, within mature TLS (40) and that Tfh cells play a crucial role in promoting the formation of GCs and orchestrating the differentiation of GC B cells, thereby shaping the anti-tumor TIME (41, 42). Consistent with these observations, ICI therapy has been shown to activate Tfh and B cells, producing tumor-reactive antibodies in murine breast and colon tumor models (123, 124). These studies underscore the indispensable role of Tfh cells in facilitating the GC response within mature TLS.

### 5.5 Mature TLS are vital sites for lymphocyte communication, promoting the activation and proliferation of tumor-reactive CD8+ T cells

Activated CD8+ T cells exert anti-tumor functions via cytolytic molecules such as granzyme and granzyme lysin or inflammatory cytokines such as IFN-γ and TNF-α (32). Immunofluorescence staining images of TLS derived from various solid tumors consistently demonstrate a relatively low presence of infiltrating CD8+ T cells within the TLS, with CD8+ effector memory T cells predominantly located at the TLS periphery (31). Nonetheless, the number of CD8+ T cells infiltrating the tumor stroma outside of mature TLS increases due to the tumor-killing activity of CD8+ T cells. The favorable clinical prognosis associated with mature TLS is likely closely linked to the presence and activity of CD8+ T cells.

Studies indicate a higher presence of CD8+ T cells within mature TLS compared to immature TLS, with the density of intra-tumoral CD8+ T cells associated with the fraction of SFL-TLS. This correlation suggests a potential link between TLS maturation and the infiltration of cytotoxic T lymphocytes (CTLs) into the tumor core. Moreover, mature TLS are identified as an independent low-risk factor for lymph node metastasis, possibly due to the heightened frequency of cytotoxic lymphocyte infiltration in TDLNs within the mature TLS group. It is hypothesized that CTLs originating from mature TLS within tumors migrate to TDLNs through lymphatic vessels, thereby preventing lymph node metastasis (30, 125). Researchers also found that HEV mediated the migration of lymphocytes, particularly CD8+ T cells, from peripheral tissues to tumor locations treated with combination immunotherapy and improved patient survival prognosis (126). Additional studies have shown increased infiltration of CD8+ T cells in the tumor stroma of TLS-enriched soft tissue sarcomas. Depletion of these T cells could provide insights into the relationship between immune checkpoint therapy and TLS, elucidating why checkpoint inhibitors may elicit robust anti-tumor immune responses in TLS-enriched tumor environments (26). In esophageal squamous carcinoma, mature TLS exhibit a heightened presence of CD8+ T cells compared to immature TLS, yet a concurrent reduction in the expression of MHC class I molecules within mature TLS is accompanied by an increase in CD4+ Th17 cells, suggesting that Th17 cells may augment CD8+ T-cell immunity beyond the confines of TLS within tumor tissues (33, 127). In summary, while some studies indicate a correlation between mature TLS and elevated CD8+ T cells within the TLS themself, mature TLS are also associated with an enrichment of tumor-infiltrating CD8+ T cells outside the TLS. Thus, it is imperative to assess whether the survival disparities observed in TLS-positive tumors due to CD8+ T-cell density originate solely from the TLS or reflect the overall CD8+ T-cell density within the TME.

## 6 New therapeutic strategy: induction of mature tertiary lymphoid structure generation

Given the robust immune activity associated with mature TLS, the induction of their generation emerges as a pivotal strategy in cancer treatment. Numerous studies have demonstrated that anti-PD-1 drugs augment the activation of Tfh cells on B cells, thereby promoting the formation and maturation of TLS in tumor tissues. Consequently, patients subjected to immunotherapy often exhibit improved clinical outcomes, potentially attributed to the substantial infiltration of IgG antibodies within mature TLS (117, 128). The development and maturation of TLS hinge upon the recruitment of immune cells facilitated by various chemokines. Hence, the exogenous provision or augmentation of pre-existing chemokine secretion represents a promising approach for inducing mature TLS formation. Notably, lysogenic virus therapy has been shown to increase cytokine and chemokine levels in the TME, thereby fostering the recruitment of immune cells to mature TLS sites (129). Tfh cells and CXCL13 play pivotal roles in the formation of mature TLS, particularly in the establishment and maintenance of GCs. GCs have been associated with a balance between Tfh cells and follicular regulatory T (Tfr) cells within TLS, with a more robust Tfh cell activity favoring TLS maturation (37, 130). Additionally, TLS formation can be induced by intra-tumoral infusion of autologous Tfh cells, which secrete LIGHT, IL-21, and CXCL13. These factors contribute to the formation of HEVs, with CXCL13 attracting B cells to migrate to the tumor region and IL-21 supporting recruited B cells. Collectively, these mechanisms encourage the spontaneous formation and maturation of TLS in ovarian cancer (131).

Although the laboratory demonstrations of methods for inducing TLS maturation are promising, their translation into clinical practice is still in the preliminary exploration phase. Various challenges, including potential toxicity, lengthy formation cycles, and uncontrolled TLS emergence (132, 133)[159, 160], constrain the clinical applicability of these approaches. Nevertheless, innovative therapeutic strategies aimed at *de novo* induction of mature TLS offer significant potential for enhancing outcomes in patients with cancer. These research advancements may herald a new era in cancer immunotherapy, ultimately leading to improved patient outcomes and survival rates.

## 7 Conclusion and outlook

Recent research highlighting the prognostic and predictive significance of TLS in cancer has sparked considerable interest in studying TLS. The augmented immune response associated with TLS maturity may significantly contribute to the favorable prognosis observed in cancer immunotherapy. Mature TLS are preferential sites for antigen presentation, T-cell and B-cell activation and differentiation, and antibody production, facilitating localized induction and maintenance of the anti-tumor immune response, correlating with favorable immunotherapy outcomes. However, our current understanding of the mechanisms by which mature TLS control tumor growth and predict checkpoint blockade responses remains limited.

The requirement for comprehensive and in-depth research on TLS deserves considerable attention; based on this review, the authors posit that forthcoming research related to TLS should prioritize the following areas. Firstly, the intricate pathways of action and interactions among cells within the TLS, particularly the specific functions of B cells in mature TLS and the targets and nature of B-cell antibody antigens, still need to be fully elucidated. Therefore, further in-depth studies are warranted to gain insights into the role of mature TLS and B cells in the immune response against tumors. Moreover, although we can speculate potential maturation mechanisms of TLS from established processes in lymphoid follicles, further investigation is imperative to characterize mature TLS in patients with cancer accurately. This necessity arises from notable disparities between lymph nodes and TLS, compounded by the predominant reliance on noncancerous animal models in current studies examining TLS formation mechanisms. Additionally, varying perspectives across studies exist regarding the classification of PFL-TLS as mature TLS; inconsistent criteria for evaluating TLS maturity will impede the advancement of TLS-related research. Recent efforts have attempted to categorize TLS maturity status in patients into grades, yet the oversimplified classification solely based on the presence or absence of mature TLS overlooks the potential influence of varying TLS morphologies and their proportions on maturity levels; the current classification system needs to improve precision (35). Finally, we summarized various methodologies for inducing the formation of mature TLS, encompassing chemotherapy, immunotherapy, cell infusion, and alternative strategies. Nonetheless, some existing difficulties limit the clinical application of the above approaches. Hence, there is a pressing need to advance safer and more dependable TLS induction modalities. While the significance of TLS in cancer immunotherapy is widely acknowledged, numerous unresolved inquiries and obstacles persist. Through meticulous research and innovative methodologies, researchers are currently addressing the ongoing challenge of optimizing the application of TLS in cancer therapy.

## Author contributions

XB: Writing – original draft, Writing – review & editing. XL: Writing – original draft, Writing – review & editing. MX: Software, Visualization, Writing – original draft. JY: Software, Visualization, Writing – review & editing. JS: Software, Visualization, Writing – original draft. XM: Project administration, Resources, Writing – original draft. XZ: Software, Supervision, Writing – review & editing. YZ: Software, Supervision, Writing – review & editing. YML: Software, Supervision, Writing – review & editing. WH: Software, Supervision, Project administration, Writing – original draft. YRL: Software, Supervision, Writing – review & editing. HH: Writing – review & editing, Supervision, Project administration. LX: Writing – review & editing, Supervision, Project administration. XX: Funding acquisition, Resources, Writing – original draft, Writing – review & editing.

## Glossary

**Table:**

TLS	Tertiary lymphoid structures
ICI	Immune checkpoint inhibitor
NSCLC	Non-small cell lung cancer
PFS	Progression-free survival
OS	Overall survival
TMB	Tumor mutational burden
PD-1	Programmed death-1
PD-L1	Programmed death-ligand 1
GC	Germinal center
TRM	Tissue-resident memory T cells
HEV	High endothelial venule
DC	Dendritic cell
FDC	Follicular dendritic cell
E-TLS	Early TLS
PFL-TLS	Primary follicle-like TLS
SFL-TLS	Secondary follicle-like TLS
BCRs	B-cell receptors
TIL-B	Tumor-infiltrating B cell
APCs	Antigen-presenting cells
GZMB	Granzyme B
ORRs	Objective response rates
Tfhs	Follicular helper T cells
SHM	Somatic hypermutation
CSR	Class switch recombination
ADCP	Antibody-dependent cellular phagocytosis
ADCC	Antibody-dependent cytotoxicity
CDC	Complement-dependent cytotoxicity
ICs	Immune complexes
PC	Plasma cell
LTi cells	Lymphoid tissue-inducing cells
LTα1β2	Lymphotoxin α1β2
LTβR	Lymphotoxin β
CAFs	Cancer-associated fibroblasts
LTo cells	Lymphoid tissue organizer cells
VEGFC	Vascular endothelial growth factor C
FRCs	Fibroblastic reticular cells
SLOs	Secondary lymphoid organs
TDLNs	Tumor-draining lymph nodes
NK cell	Natural killer cell
AID	Activation-induced cytidine deaminase
MHC	Major histocompatibility complex
TILs	Tumor-infiltrating lymphocytes
PIGR	Polymeric immunoglobulin receptor
IgA	Immunoglobulin A
FcγRIII	Fcγ
TCGA	Cancer Genome Atlas
TIME	Tumor immune microenvironment
CTLs	Cytotoxic T lymphocytes
Tfr cells	Follicular regulatory T cells
TME	Tumor microenvironment
TAA	Tumor-associated antigen
TAB	Tumor-associated B cell
HPV	Human papillomavirus.
